# COVID-19 Testing Practices, Preventive Behaviors, and Factors Associated With Test Positivity: Population-Based Statewide Survey Study

**DOI:** 10.2196/34579

**Published:** 2023-04-19

**Authors:** Mufaro Kanyangarara, Virginie Daguise, Lídia Gual-Gonzalez, Alain H Litwin, Jeffrey Korte, Connor Ross, Melissa S Nolan, SC STRONG Project Team

**Affiliations:** 1 Department of Epidemiology and Biostatistics University of South Carolina Arnold School of Public Health Columbia, SC United States; 2 South Carolina Department of Health and Environmental Control Columbia, SC United States; 3 University of South Carolina School of Medicine Greenville, SC United States; 4 Prisma Health Greenville, SC United States; 5 Clemson University Clemson, SC United States; 6 Department of Public Health Sciences Medical University of South Carolina Charleston, SC United States; 7 See Acknowledgements Columbia, SC United States

**Keywords:** SARS-CoV-2, South Carolina, surveillance, attitude, behavior, COVID-19, testing, prevention, United States, population, survey, risk, perception, risk factor

## Abstract

**Background:**

The COVID-19 pandemic has challenged public health efforts globally. Timely population-based surveillance is crucial to support public health programs and policies to limit the spread of COVID-19. The South Carolina (SC) Sampling and Testing Representative Outreach for Novel Coronavirus Guidance (SC STRONG) statewide initiative was established to estimate population-level prevalence and immunity and characterize the transmission dynamics of SARS-CoV-2 using community testing and online surveys.

**Objective:**

This paper aimed to leverage the survey data collected as part of the initiative to understand risk perceptions, testing practices, and preventive behaviors and identify risk factors for COVID-19 test positivity in SC over time.

**Methods:**

Probability proportionate to size cluster random sampling was used to select SC residents to participate in testing for COVID-19 infection and antibodies and to complete an online survey. This paper focuses on data from the online surveys completed between November 2020 and June 2021. Descriptive statistics were used to describe risk perceptions, attitudes and behaviors, and associated changes over time. Univariate and multivariate logistic regression models were used to identify factors associated with self-reported COVID-19 test positivity.

**Results:**

Among the 7170 online survey respondents, 58.7% (4213/7170) self-reported ever testing for COVID-19. The most commonly cited barriers to testing were inconvenient dates, time, and location, as well as discomfort. Overall, 18.7% (790/7170) of respondents reported a history of COVID-19 test positivity. Multivariate logistic regression results indicated that individuals who were aged 50 years or older, self-identified as Black/African American, were obese, and were employed as frontline health care workers or nursing home staff were more likely to self-report COVID-19 test positivity. By contrast, there was a decreased likelihood of test positivity among respondents who were concerned about the burden of COVID-19 in their community and about being infected.

**Conclusions:**

Strategies to remove testing barriers should be implemented to improve access. Our findings provide insights on statewide testing patterns, adoption of prevention behaviors, and risk factors for infection and may inform public health strategies to curb transmission.

## Introduction

The year 2020 brought new public health challenges and highlighted the need for data to inform ongoing public health response efforts. In South Carolina (SC), the first case of COVID-19 was detected on March 4, 2020 [1]. As of July 2021, the cumulative numbers of confirmed COVID-19 cases and associated deaths in SC were over 700,000 and 11,500, respectively [1]. Mitigation measures in the state have included face mask ordinances, closure of nonessential businesses, isolation of COVID-19 cases, tracing and quarantining of close contacts of cases, and promotion of personal protective behaviors, such as routine testing, face coverings, hand hygiene, and social distancing. These measures were implemented to reduce disease spread and remain crucial to prevent overcrowding hospitals and emergency rooms with patients with severe disease during local outbreaks [2]. Even as COVID-19 vaccines are rolled out, preventive behaviors remain crucial components in the arsenal to address the pandemic. To effectively minimize COVID-19 transmission risk and prevent the loss of life, achieving and sustaining high uptake of preventive behaviors needs to be prioritized [3,4].

The adoption of preventive behaviors in part depends on the perceived severity of disease, perceived susceptibility to disease, benefits of compliance, and removal of barriers to the adoption of protective behaviors [5]. Knowledge and attitudes partially influence the adoption of these behaviors [6]. While several studies have assessed COVID-19 attitudes and behaviors, most were at the national scale or based on convenience sampling [7-11]. Some of these studies discuss the role of information exposure and knowledge in health behavior, and national-level estimates are likely to differ from state-level estimates. There is a paucity of research at the state level, particularly in SC, a state primarily defined by conservative ideology; thus, a description of the population’s perceptions is warranted for a locally tailored public health response [12-14]. Given the continuing incidence of COVID-19, the overwhelming public health burden, and the rapidly changing epidemiology of COVID-19, there is an urgent need for timely population-based surveillance data to support state-specific, targeted policies and public health efforts to address the pandemic.

The objectives of this analysis were 2-fold: first, to understand SC respondents’ testing patterns, risk perceptions, and preventive behaviors and associated temporal changes and second, to identify risk factors for COVID-19 test positivity to inform public health policies and programs in SC. Understanding the population’s perceived risk allows for the targeting of public health interventions to reduce the burden of COVID-19 while addressing health inequities [15]. This paper reports on results from 3 rounds of surveys conducted from November 2020 to June 2021 among 7170 SC residents participating in the SC Sampling and Testing Representative Outreach for Novel Coronavirus Guidance (SC STRONG) project. The SC STRONG project was established in October 2020 across all 4 public health regions in the state with the goal of estimating population-level seropositivity and immunity and characterizing population-specific transmission dynamics [16]. This ongoing initiative is led by the SC Department of Health and Environmental Control (SC DHEC) together with multidisciplinary academic collaborators, local health clinics, and health care providers across the state. The project consists of multi-round cross-sectional community testing and surveys, and to date, 3 rounds of community testing and surveys have been completed. The project complements reportable disease surveillance for COVID-19 by characterizing attitudes and behaviors toward public health measures to limit COVID-19 transmission among a large random sample of SC residents.

## Methods

### Setting and Study Design

**Figure 1:**
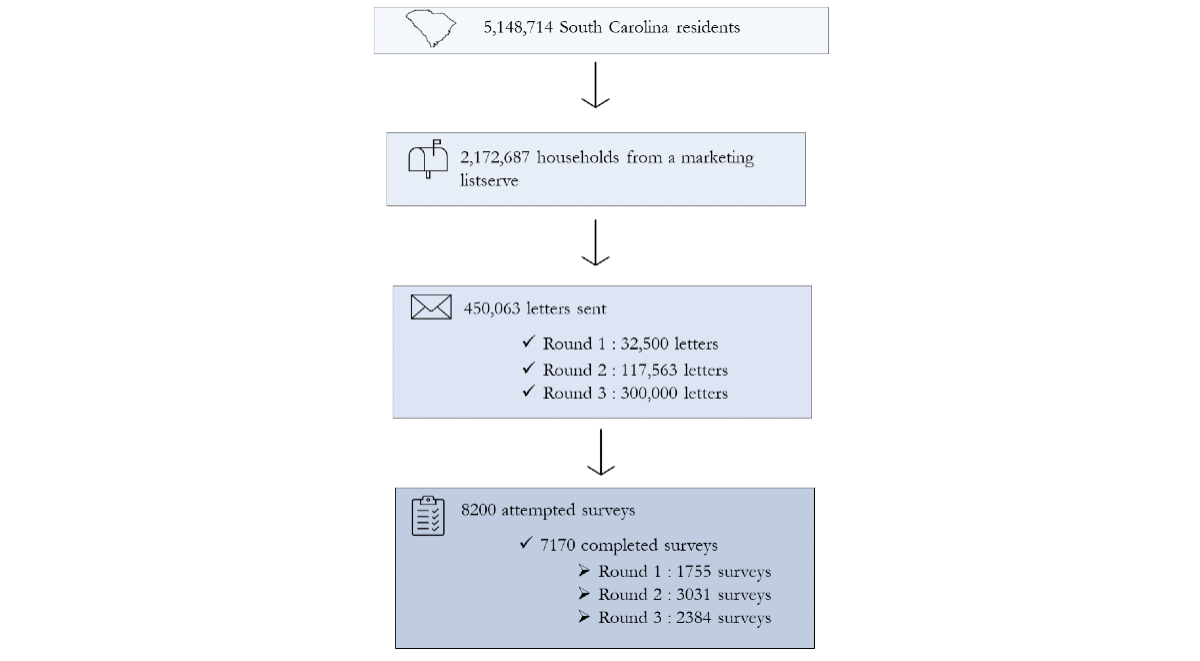
Flow diagram of study participation.

SC has a population of approximately 4.86 million inhabitants aged 5 years and older [17]. The state has 46 counties distributed across 4 public health regions, all of which were included in the SC STRONG project. A description of the SC STRONG project has been published elsewhere [18]. Briefly, a direct mailer marketing list with 2,172,687 unique physical addresses was purchased from Mailers Haven and used to establish a sampling frame (Figure 1). Multi-stage cluster sampling with probability proportionate to size was used to select clusters and 30 residents from each selected cluster. Invitation letters to participate in testing for active COVID-19 infection and antibodies and to complete an online survey were mailed to selected residents. The invitation letters included an explanation of the project, instructions on how to participate in free community testing, and a QR code and URL for the online electronic survey. The number of invitations mailed each round varied depending on the anticipated response rate. To improve response rates, at least two reminders were sent to selected residents. Additionally, to accommodate participants with no internet access and allow them to complete the online survey, the testing site provided the option to fill out the survey on site.

The online survey was administered via REDCap (Research Electronic Data Capture) and hosted by Health Sciences South Carolina. REDCap is a secure web-based software platform designed to support data capture for research studies [19]. A standardized survey questionnaire available in English and Spanish was used to collect data on sociodemographic characteristics, testing behaviors, daily behaviors, and risk perceptions. The questionnaire consisted of questions from existing COVID-19 surveys and new questions developed by the SC STRONG team [20]. Additional survey questions regarding acceptance, motivations, and barriers to getting the COVID-19 vaccine were added in January 2021.

### Statistical Analysis

Descriptive statistics and visualizations were used to describe the sample population and chi-square statistics were used to compare sociodemographic characteristics of the SC general population, survey respondents, and respondents who reported ever testing for COVID-19. Data on the SC general population were obtained from the 2019 American Community Survey [17]. Testing behaviors and barriers, attitudes and barriers toward the vaccine, and uptake of preventive behaviors were summarized. The preventive behaviors considered included practicing social distancing, wearing a mask, and self-isolation or quarantining. Respondents were considered adherent to preventive behaviors if they reported practicing these behaviors “sometimes,” “most of the time,” or “all the time” during the previous 2 weeks. Univariate logistic regression models with “week” as an independent variable were used to assess temporal changes in behaviors and attitudes. Changes in behaviors and attitudes per week were expressed as maximum likelihood estimates with 95% CIs.

History of testing was derived from 2 survey questions: “Have you ever been tested for active coronavirus infection (nasal or saliva test)?” and “Have you ever been tested for coronavirus antibodies (blood test)?” As the goal was to identify risk factors for a positive COVID-19 test among those with history of testing, individuals with no prior testing for active coronavirus infection or antibodies were excluded from this analysis. The outcome of interest was based on self-reported diagnosis of coronavirus antibodies or active coronavirus infection derived from the following survey questions: “Have you ever tested positive for active coronavirus infection?” and “Have you ever tested positive for coronavirus antibodies?” Respondents who answered yes to either question were categorized as having ever tested positive for COVID-19. The likelihood of COVID-19 test positivity was assessed with univariate multivariable logistic regression. Explanatory variables of interest were selected based on previous studies assessing risk factors for COVID-19. To account for likely racial inequalities, “Black” and “Hispanic” were added as indicator variables. Household income was added as a proxy for socioeconomic status. Other explanatory variables considered were preexisting medical conditions, number of household members, living or working in congregate settings, testing behaviors, risk perceptions, and knowledge of COVID-19 preventive measures. To account for differences in risk of COVID-19 over time, models adjusted for time in weeks since recruitment for SC STRONG began. In addition to age, gender, income, and race or ethnicity, which were specified a priori, variables with a *P* value less than 0.1 in the univariate logistic regression models were included in the initial multivariable regression model. A stepwise backwards elimination procedure was used to obtain the final multivariable model. Crude and adjusted odds ratios (aORs) from the logistic regression analyses are reported. Checks for multicollinearity were performed using variance inflation factors (VIFs). Covariates with a VIF greater than 8 were dropped. A *P* value less than .05 was considered statistically significant. Analyses were conducted using STATA/SE (version 16.1; StataCorp) and SAS Studio (version 3.8; SAS Institute).

### Ethical Considerations

Human-subjects ethics approval was sought for the SC STRONG project. The institutional review boards at the University of South Carolina and the SC DHEC both determined that SC STRONG project activities did not constitute human-subjects research under the auspices of public health surveillance (102072). Participation in testing and survey completion was voluntary.

## Results

### Overview

**Figure 2:**
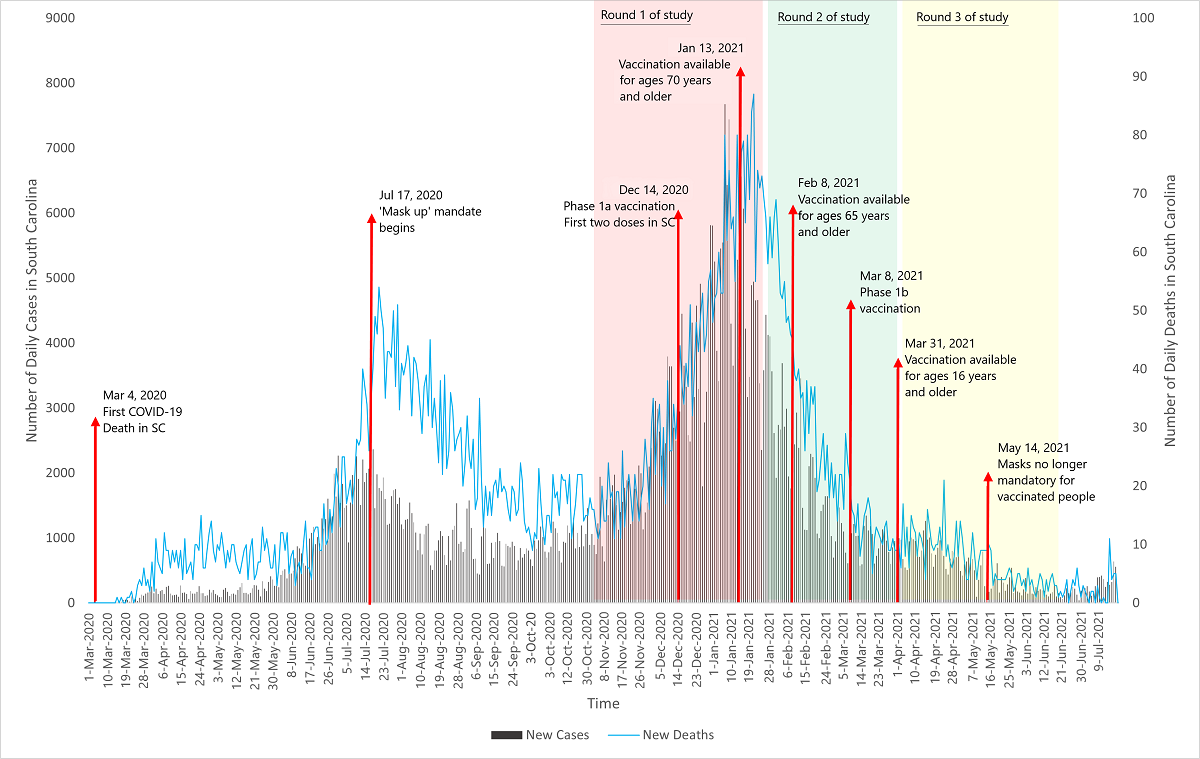
Trends in number of COVID-19 cases and deaths in South Carolina, public health measures, and data collection rounds. SC: South Carolina.

**Figure 3:**
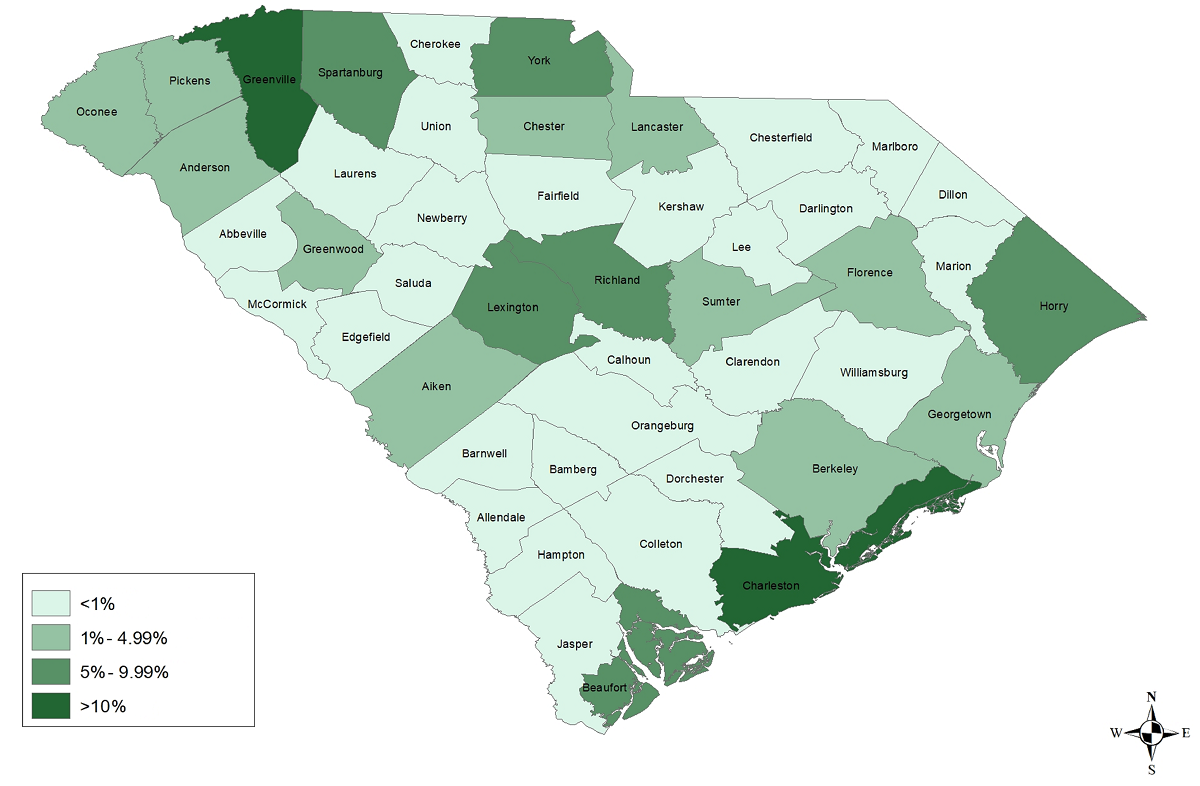
Percentage of responses by county.

From October 26, 2020, to June 16, 2021, 3 rounds of data collection were conducted, and 450,063 invitation letters were mailed out (Figure 1). The first round of data collection took place from October 26, 2020, to January 22, 2021, and 32,500 invitation letters were mailed. This round coincided with the second wave, when daily COVID-19 cases peaked to over 5500 (Figure 2). The second round of data collection took place from January 25, 2021, to March 31, 2021, and 117,563 letters were mailed (Figure 1). This round was toward the end of the second wave of COVID-19, when the daily number of cases fell to less than 1200 per day and COVID-19 vaccines became available under phase 1b to populations living and working in shared settings with increased risk and to frontline essential workers (Figure 2). The third round of data collection ran from April 2, 2021, to June 16, 2021, and 300,000 letters were mailed (Figure 1). At the time of this round, COVID-19 vaccines were available to individuals aged 16 years or older (Figure 3).

A total of 8200 surveys were attempted across the 3 rounds, and 1030 surveys were excluded due to incomplete or inconsistent responses; therefore, a total of 7170 completed surveys was used for the present analysis (Figure 1). The response rate for all 3 rounds was 1.6% (7170/450,063). The response rate decreased with each round, from 5.4% (1755/32,500) in the first round to 0.8% (2384/300,000) in the third round. There was also variation in the percentage of responses by county of residence (Figure 3). More survey respondents lived in the upstate region (eg, Greenville County at 950/7091, 13.4%), in coastal areas (eg, Charleston County at 726/7091, 10.3%, and Horry County at 639/7091, 9%), and in areas surrounding the capital city (eg, Lexington County at 500/7091, 7.1%, and Richland County at 667/7091, 9.4%).

Most survey respondents were aged 60 years or older (4087/7170, 57%), female (4151/7170, 57.9%), and Caucasian (6087/7170, 84.9%; Table 1). Survey respondents were fairly representative of the general SC population in terms of gender and income. However, Hispanic/Latino Americans and African Americans were underrepresented in the survey. Whereas 5.8% and 26.3% of the general SC population are Hispanic/Latino Americans and African Americans, only 2.2% (158/7170) and 8.9% (683/7170) of survey respondents identified as Hispanic/Latino Americans and African Americans, respectively. Moreover, older age groups were overrepresented in the study population. There were also differences among survey respondents by COVID-19 testing status. Respondents who were previously tested for COVID-19 tended to be younger, female, and have higher income than the overall sample of survey respondents (Table 1).

**Table 1:** Sociodemographic characteristics of survey respondents (n=7170) and respondents ever tested for COVID-19 (n=4197).

Characteristics			General South Carolina population^a^ (N=5,148,714), %		Survey respondents (n=7170), n (%)		*P* value^b^			Ever tested for COVID-19 (n=4197), n (%)			*P* value^c^	
**Age (years)**								<.001						<.001
	<18	21.6		122 (1.7)					0 (0)					
	18-29	15.9		194 (2.7)					201 (4.8)					
	30-39	12.6		445 (6.2)					290 (6.9)					
	40-49	12		882 (12.3)					546 (13)					
	50-59	13		1441 (20.1)					894 (21.3)					
	60-69	12.6		2251 (31.4)					1267 (30.2)					
	≥70	12.3		1836 (25.6)					999 (23.8)					
**Gender**								.40						<.001
	Female	51.7		4151 (57.9)					2514 (59.9)					
	Male	48.3		2976 (41.5)					1683 (40.1)					
	Other	0		43 (0.6)					0 (0)					
**Race/ethnicity^d^**								.007						
	Hispanic/Latino American	5.8		158 (2.2)					92 (2.2)			.80		
	African American	26.3		638 (8.9)					436 (10.4)			.10		
	Caucasian	63.6		6087 (84.9)					3542 (84.4)			.30		
	Asian	1.7		129 (1.8)					55 (1.3)			<.001		
	Native American	0.3		50 (0.7)					29 (0.7)			.90		
	Other/prefer not to answer	0.2		222 (3.1)					25 (0.6)			.20		
**Income (US $)**								.20						<.001
	≤14,999	11.5		208 (2.9)					109 (2.6)					
	15,000-34,999	19.1		595 (8.3)					327 (7.8)					
	35,000-49,999	13.9		638 (8.9)					378 (9)					
	50,000-74,999	18.2		1097 (15.3)					646 (15.4)					
	75,000-99,999	12.6		939 (13.1)					571 (13.6)					
	100,000-149,000	13.6		1111 (15.5)					638 (15.2)					
	≥150,000	11		968 (13.5)					613 (14.6)					

^a^Data obtained from South Carolina Department of Health and Environmental Control [16].

^b^*P* values (chi square test) for differences between the South Carolina general population and the survey respondents.

^c^*P* values (chi square test) for differences between those ever tested for COVID-19 and those with no history of testing.

^d^Percentages may add to more than 100%, as survey respondents could give multiple answers.

### Impact of the Pandemic

The COVID-19 pandemic impacted the lives of SC residents in multiple ways. In terms of employment, 3.8% (271/7170) reported becoming unemployed during the pandemic, 10.6% (760/7170) reported reductions in income or pay, and 7.8% (562/7170) reported working fewer hours than normal (Table 2). In terms of mental and emotional health, 72.1% (5173/7170) reported often feeling stressed, nervous, or on the edge, 56.7% (4063/7170) reported often feeling sad or depressed in the previous 2 weeks, and 19% (1365/7170) reported having physical reactions when thinking about the pandemic. However, only 2.6% (191/7170) reported being diagnosed with anxiety or depression during the pandemic. In terms of food security, very few respondents reported experiencing lack of food (73/7170, 1%) or going without eating (282/7170, 4%) in the previous 2 weeks due to lack of money or resources.

**Table 2:** Trends in behaviors and attitudes among South Carolina residents toward testing, vaccination, and preventive measures for the COVID-19 pandemic (n=7170).

Behaviors and attitudes		Respondents, n (%)	Change per week^a^	*P* value	
Ever been tested		4213 (58.7)	0.03	<.001	
Active infection^b^		3988 (94.6)	0.03	<.001	
Antibodies^b^		862 (20.5)	0.02	<.001	
Ever tested positive^b^		790 (18.8)	0.03	<.001	
**Reasons for testing^b,c^**					<.001
	Close contact tested positive	1397 (33.2)	0.02	<.001	
	Coronavirus-like symptoms	1314 (31.2)	0.01	<.001	
	Concerned about high number of cases in community	789 (18.7)	0.02	<.001	
	Medical, travel, or employment requirement	834 (19.7)	0.02	<.001	
	Curiosity	721 (17.1)	0.02	<.001	
**Testing location^b^**					<.001
	Doctor’s office	943 (22.3)	0.01	<.001	
	Drive through or community pop-up	2746 (65.2)	0.02	<.001	
**Frequency of COVID–19 testing^b^**					
	1 time	1841 (43.7)	–0.03	<.001	
	2-5 times	2167 (51.4)	0.02	<.001	
	6 or more times	205 (4.9)	0.03	<.001	
**Barriers to testing^c,d^**					
	Inconvenient dates and times	149 (20.3)	–0.03	.002	
	Discomfort of the test	141 (19.2)	–0.03	<.001	
	Did not know where to go	118 (16.1)	–0.07	<.001	
	Economic	98 (13.4)	–0.03	.007	
	Inconvenient location	87 (11.9)	–0.03	.01	
**Attitudes toward vaccines^c^**					
	Vaccinated	2498 (45.8)	0.20	<.001	
	Think vaccines are safe	3982 (72.9)	0.02	<.001	
	Plan to get vaccinated	2270 (41.6)	–0.16	<.001	
	No plan to get vaccinated	516 (9.5)	N/A^e^		
**Barriers to vaccination^c^**					
	Fear of needles	24 (4.2)	–^f^	.9	
	Uncomfortable being one of the first vaccinated	282 (49.4)	–^f^	.06	
**Practice of preventive behaviors**					
	Social distancing	7068 (98.6)	–0.1	<.001	
	Mask wearing	7035 (98.1)	–0.07	<.001	
	Self–isolation/quarantine	676 (9.4)	–0.04	<.001	
**Impact of pandemic on work**					
	Became unemployed	271 (3.8)	–0.06	<.001	
	Pay was reduced	760 (10.6)	–0.07	<.001	
	Worked with children at home	455 (6.3)	–0.07	<.001	
	Worked less hours	562 (7.8)	–0.08	<.001	
	Worked from home more	1469 (20.5)	–0.08	<.001	
**Impact of pandemic on mental health**					
	Being sad/depressed in the past 2 weeks	4063 (56.7)	–0.02	<.001	
	Being diagnosed with depression or anxiety	191 (2.6)	N/A^e^		
	Having felt physical reactions when thinking about the pandemic in the past 2 weeks	1365 (19)	–0.02	<.001	
	Having felt some level of stress, anxiety, or being on the edge in the past 2 weeks	5173 (72.1)	–0.03	<.001	
**Impact of pandemic on food insecurity**					
	Lack of food in the past 2 weeks	73 (1)	–^f^	.1	
	Gone without eating in the past 2 weeks	282 (4)	–0.02	<.001	

^a^Adjusted logistic regressions including time (in weeks) as a predictor to assess for trends over time.

^b^Percentages represent percentages of those ever tested.

^c^Percentage may add more to than 100%, as survey respondents could give multiple answers.

^d^Percentage of those with no history of testing (n=593).

^e^N/A: not applicable, as the variable violated the assumption for logistic regression.

^f^Point estimate for the change per week was smaller than 0.001; the – symbol indicates the direction of the change (ie, negative).

### COVID-19 Testing Practices and Barriers

Of the 7170 survey respondents, 58.7% (4213/7170) reported ever testing for COVID-19 active infection or antibodies, and there was a 3% increased likelihood of testing for COVID-19 with every week that passed (Table 2). The most common barriers to testing were inconvenient times and dates (143/593, 20.3%), the discomfort of the test (141/593, 19.2%), and lack of knowledge of where to go to be tested (118/593, 16.1%). Nevertheless, the likelihood of citing these testing barriers decreased with time. On the other hand, the most common reasons for getting tested were having a close contact who had tested positive (1397/4213, 33.2%); having coronavirus-like symptoms (1314/4213, 31.2%); medical, travel, or employment requirements (834/4213, 19.8%); and being concerned about the high number of COVID-19 cases in the community (789/4213, 18.7%). A less common reason for testing was curiosity (721/4213, 17.1%). More than half of respondents received testing at a drive-through or community pop-up testing site (2746/4213, 65.2%) and reported being tested more than once (2372/4213, 56.3%). Over time, respondents were more likely to get tested and reported getting tested in a drive-through or community pop-up testing site. Of those who had ever been tested, 18.8% (790/4213) reported testing positive for antibodies or active infection at some point.

### COVID-19 Preventive Behaviors

Most respondents reported social distancing (7068/7170, 98.6%) and wearing face masks (7035/7170, 98.1%) in the previous 2 weeks, and fewer reported self-isolation (676/7170, 9.4%) or quarantining (Table 2). The likelihood of engaging in these practices decreased with time (*P*<.001). Overall, 45.8% (2498/5460) of respondents reported being vaccinated against COVID-19. An assessment of attitudes toward the vaccine indicated that 72.9% (3982/5460) of respondents thought the COVID-19 vaccines were safe, and 41.6% (2270/5460) planned to get vaccinated at some point. Among those who reported not planning to get the vaccine, the main reason was discomfort with being one of the first to be vaccinated (282/571, 49.4%). Overall, there was an increased likelihood of vaccination (20%) and positive attitudes toward the vaccines per week.

### Factors Associated With COVID-19 Test Positivity

In the multivariable logistic regression analysis, several factors were significantly associated with COVID-19 test positivity (Table 2). The likelihood of COVID-19 test positivity was increased among individuals who identified as Black/African American, were employed in a nursing home or as a frontline health care worker, were obese, and reported testing at the doctor’s office. Furthermore, the aOR of a positive COVID-19 test was highest for individuals who decided to test because of coronavirus-like symptoms (aOR 7.37, 95% CI 6.08-8.94) and those with a family member or close friend who currently or previously had COVID-19 (aOR 4.61, 95% CI 3.41-6.24). On the other hand, the likelihood of COVID-19 test positivity was decreased among individuals who expressed concern about being infected (aOR 0.38, aOR 0.30-0.47) and who chose to test out of concern for the perceived high burden of COVID-19 in the community (aOR 0.28, 95% CI 0.20-0.40). There was no statistically significant difference in the likelihood of test positivity by gender or income.

## Discussion

### Principal Findings

Using serial population-based surveys conducted between November 2020 and June 2021, this paper sought to describe COVID-19 risk perceptions, testing patterns, and preventive behaviors and identify factors associated with test positivity among a large random sample of SC residents. Overall, 58.7% (4213/7170) of respondents reported ever testing for COVID-19, and over time, there was a decrease in barriers to testing. This finding is consistent with the increased availability of testing locations across the state over time. Moreover, our results suggested an increased likelihood of testing over time, an increased likelihood of positivity based on symptoms and exposure, and a decreased likelihood of positivity based on individual concern. Despite increased testing, there were decreases in practicing preventive behaviors such as mask-wearing and social distancing, indicating fatigue. Previous research has shown that states with no mask policy and low mask adherence reported high COVID-19 infection rates, while states with high mask adherence and strict mask-wearing policies showed decreased infection rates [21]. It is important to note that politics has had a drastic effect on COVID-19 epidemiology, and there has been contradictory messaging regarding infection information and uptake of mitigation policies. This is reflected in misinformation driven by aligned political beliefs that conflicts with the scientific evidence and recommendations from public health agencies [22].

### Comparison With Prior Work

In this paper, 18.7% (790/7170) of respondents self-reported ever testing positive for COVID-19, which is comparable to the estimated seroprevalence of 16.4% for SC residents aged 5 years or older [18]. Our estimate could be influenced by self-selection, as our data for ever having tested positive were based on self-reports. Moreover, availability of the vaccine began in mid-January for individuals aged 70 or older, which likely also impacted testing demand. At the time of the survey, 58.7% (4213/7170) of respondents had ever been tested for COVID-19, whereas it has previously been reported that 66.7% received the vaccine, suggesting the possibility that a number of respondents did not actively seek testing due to fear of exposure [23]. Our results show the likelihood of test positivity was highest among Black/African Americans and older individuals (aged ≥50 years). Although the state does not report race or ethnicity data for testing, count data for COVID-19 deaths in SC indicate that Black/African Americans and older people are more likely to be unvaccinated, to be hospitalized for severe disease, and to die from COVID-19 [18]. The likelihood of test positivity decreased 38% in individuals who expressed concern about becoming infected, which aligns with the findings of Yildirim and Güler [24], who highlighted the direct association between perceived risk and positivity. When developing strategies to increase the adoption of preventive behaviors, understanding perceived individual risk is essential. Moreover, testing sites are often inconveniently located or open during times that are less available for people living in deep poverty due to the low flexibility of low-wage jobs and difficulty obtaining transportation. Therefore, considerable improvements should be made to accommodate the lowest socioeconomic strata, which cannot appropriately use these public health services.

### Limitations

The findings presented in this paper should be interpreted in light of several limitations. First, given the cross-sectional survey design, the temporal relationship between outcome and exposures cannot be ascertained and causality cannot be determined. Second, the representativeness of the sample is limited due to the low response rate; the lack of partner clinics in some areas of the state may have hindered participation in the community testing and online survey. Third, the sampling frame was based on a purchased marketing listserve that may not be up-to-date. Fourth, COVID-19 test positivity was based on self-reports, which are prone to bias. Fifth, comparison of sociodemographic characteristics by history of testing indicated that the respondents who sought testing were more likely to be older, female, Caucasian, and have a higher income than the general population, potentially limiting the generalizability of our results. Lastly, although an electronic survey was selected to ensure respondent safety and minimize costs of survey implementation, only about 81.8% of households in SC have access to the internet [25]. Staff were available to assist with completion of the survey online, and paper surveys were also available at the pop-up events. The analysis was restricted to responses included in the survey; this approach did not represent nonrespondents and did not capture several socioeconomic and education variables that were not included in the survey. Similarly, the results need to be considered cautiously, as the distribution of respondents might only represent individuals with broadband internet access, which would underrepresent those populations that commonly suffer health disparities and inequities related to socioeconomic, educational, and racial background.

### Conclusions

Population-based surveys are useful tools in understanding attitudes, behaviors, and practices and can inform public health responses and the adaptation of strategies to reach high-risk populations. Despite its limitations, the SC STRONG initiative represents a statewide outreach and partnership program with local clinics and health care providers. The findings presented here provide insights to understand risk perceptions and testing behaviors and have important implications given that Southern states have historically struggled with compliance and implementation of preventive public health measures.

## Abbreviations

aOR: adjusted odds ratio
DHEC: Department of Health and Environmental Control
HSSC: Health Sciences South Carolina
SC: South Carolina
SC STRONG: South Carolina Sampling and Testing Representative Outreach for Novel Coronavirus Guidance
VIF: variance inflation factor
